# Oral Verruciform Xanthoma of the Lower Lip: A Rare Entity

**DOI:** 10.7759/cureus.73352

**Published:** 2024-11-09

**Authors:** Ananjan Chatterjee, Swapan K Purkait, Ishita Banerjee, Abhishek Banerjee, Karthikeyan Ramalingam

**Affiliations:** 1 Oral and Maxillofacial Pathology, Buddha Institute of Dental Sciences and Hospital, Patna, IND; 2 Pediatric and Preventive Dentistry, Guru Nanak Institute of Dental Sciences & Research, Kolkata, IND; 3 Oral and Maxillofacial Pathology, Awadh Dental College and Hospital, Jamshedpur, IND; 4 Oral Pathology and Microbiology, Saveetha Dental College and Hospitals, Saveetha Institute of Medical and Technical Sciences, Saveetha University, Chennai, IND

**Keywords:** cathepsin b, cd68, immunohistochemistry, labial mucosa, lower lip, pathology, s100, verruciform xanthoma, verrucous pathology, xanthoma

## Abstract

Oral verruciform xanthoma (OVX) is a rare entity, and only a handful of cases have been reported in the literature to date. This innocent-looking lesion can mimic any benign epithelial or connective tissue origin neoplasm. It can present with variations in surface color and texture. Cases have been reported in intra-osseous and extra-osseous sites, mainly in masticatory mucosa and very few in non-keratinized mucosal sites. Literature suggests the xanthoma cells possess a monocyte/macrophage lineage, although the exact etiopathogenesis remains unclear. Our patient was a 20-year-old male who presented with a soft, keratotic growth in the lower lip. It clinically mimicked a mucocele or a traumatic fibroma until the mystery was resolved by histopathology and immunohistochemistry. Histopathology revealed numerous foamy, granular xanthoma cells in the stroma along with inflammation and hyperplastic epithelium. Immunohistochemistry showed positivity to CD68 and Cathepsin-B but negative to S-100. The final diagnosis was made as oral verruciform xanthoma. It was surgically excised and remained recurrence-free on follow-up.

## Introduction

Verruciform xanthoma (VX) is a hyperplastic condition of the epithelium characterized by subepithelial lipid-laden histiocyte accumulation and was first described in 1971 [1]. It occurs mainly in the gingival mucosa and can occur extraorally in anogenital and cutaneous areas. The term xanthoma originated from the Greek word xanthos, meaning yellow [2]. These lesions vary in shape and appearance from papillary, slightly raised to flat lesions. The lipid-laden macrophages are thought to accumulate beneath the epithelial surface, thus giving a pale yellowish appearance. Most VX can mimic squamous papillomas, verrucous carcinomas, or squamous cell carcinomas [3].

Histopathologically, VX is characterized by papillary proliferations of squamous epithelium showing hyperkeratosis and numerous foamy macrophages in the subepithelial areas of the stroma. The macrophagic nature of these foamy cells has been confirmed by various immunohistochemical studies using CD68, Cathepsin-B, and S-100 [4]. The etiopathogenesis of VX still remains debatable.

A literature search revealed that VX in the oral cavity predominantly involves the masticatory mucosa and tongue [1-5]. This case is special as the lower lip was involved and clinically suspected to be a mucocele. The confirmation of VX with histopathological and immunohistochemical evaluation is presented in this case report.

## Case presentation

**Figure 1:**
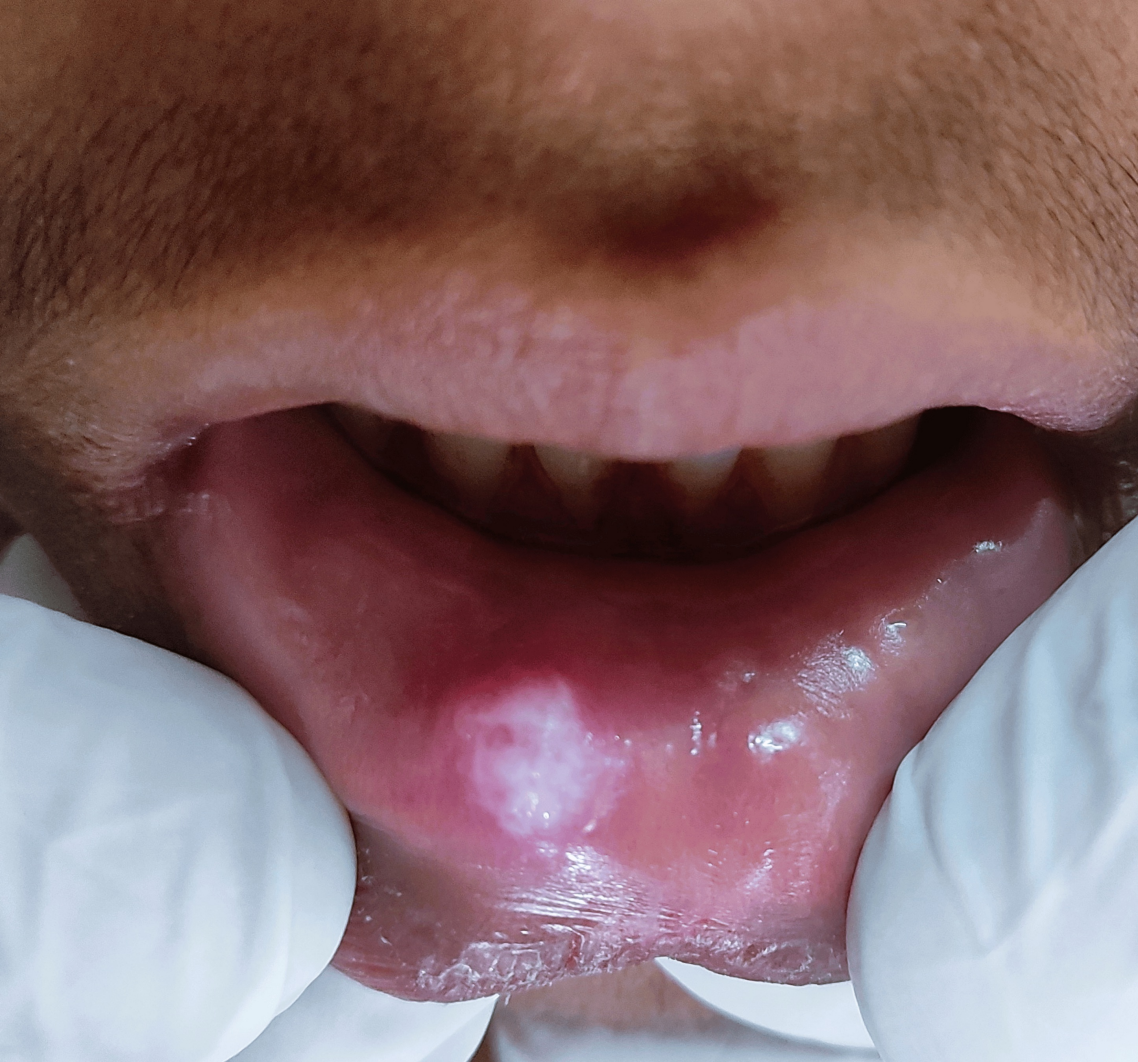
Clinical picture showing the well-circumscribed whitish lesion on the lower labial mucosa

A 20-year-old male patient reported to the outpatient department with a whitish growth on the right side of his lower lip, six months ago. There was no pain associated with the growth, but a slight difficulty during mastication and he developed a habit of biting on this growth with his upper front teeth. Past medical history, past surgical history, and past dental history were non-contributory. On intraoral examination, a growth with a whitish surface, approximately size 1.5 x 1 cm in its greatest diameter, was observed on the right side of the labial mucosa (Figure 1). It was a well-circumscribed ovoid growth with well-delineated borders, and the surrounding area was non-erythematous. On palpation, the lesion was non-tender, soft consistency in a few spots, and also firm in other areas.

**Figure 2:**
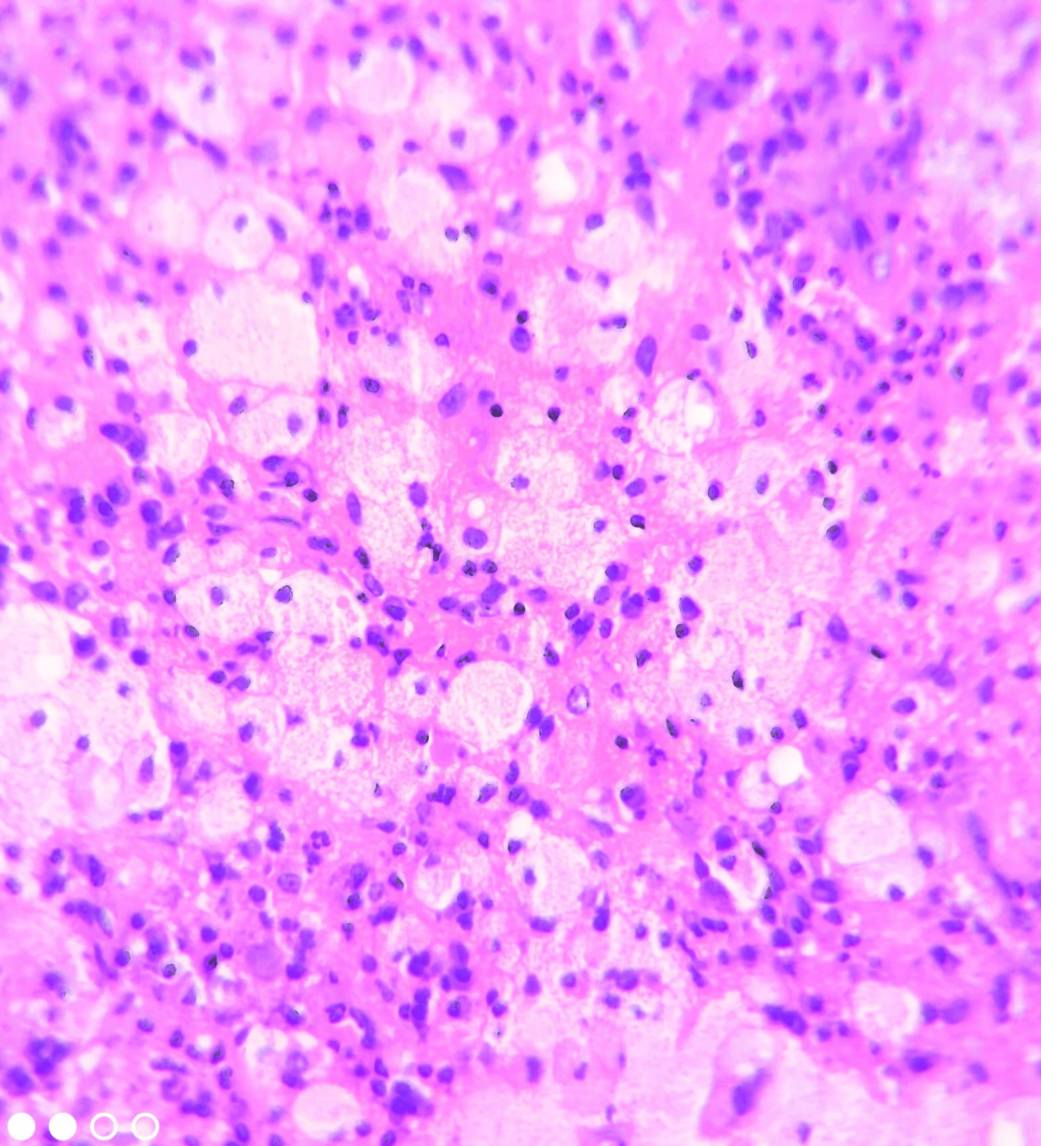
Photomicrograph showing numerous foamy xanthoma cells with pale cytoplasm and eccentric nuclei within the stroma (H&E, 20x) H&E: Hematoxylin and Eosin stain.

Based on history and clinical examination, a provisional diagnosis of mucocele was made and a differential diagnosis included traumatic fibroma. An excisional biopsy was performed under local anesthesia followed by histopathological sections which revealed stratified squamous epithelium of variable thickness overlying the connective tissue stroma with ulceration. The stromal areas showed sheets of large foamy cells with granular cytoplasm and eccentric nuclei, suggestive of foamy macrophages (Figure 2). The connective tissue stroma also showed a mild to moderate chronic inflammatory cell infiltration predominantly lymphocytes and plasma cells.

**Figure 3:**
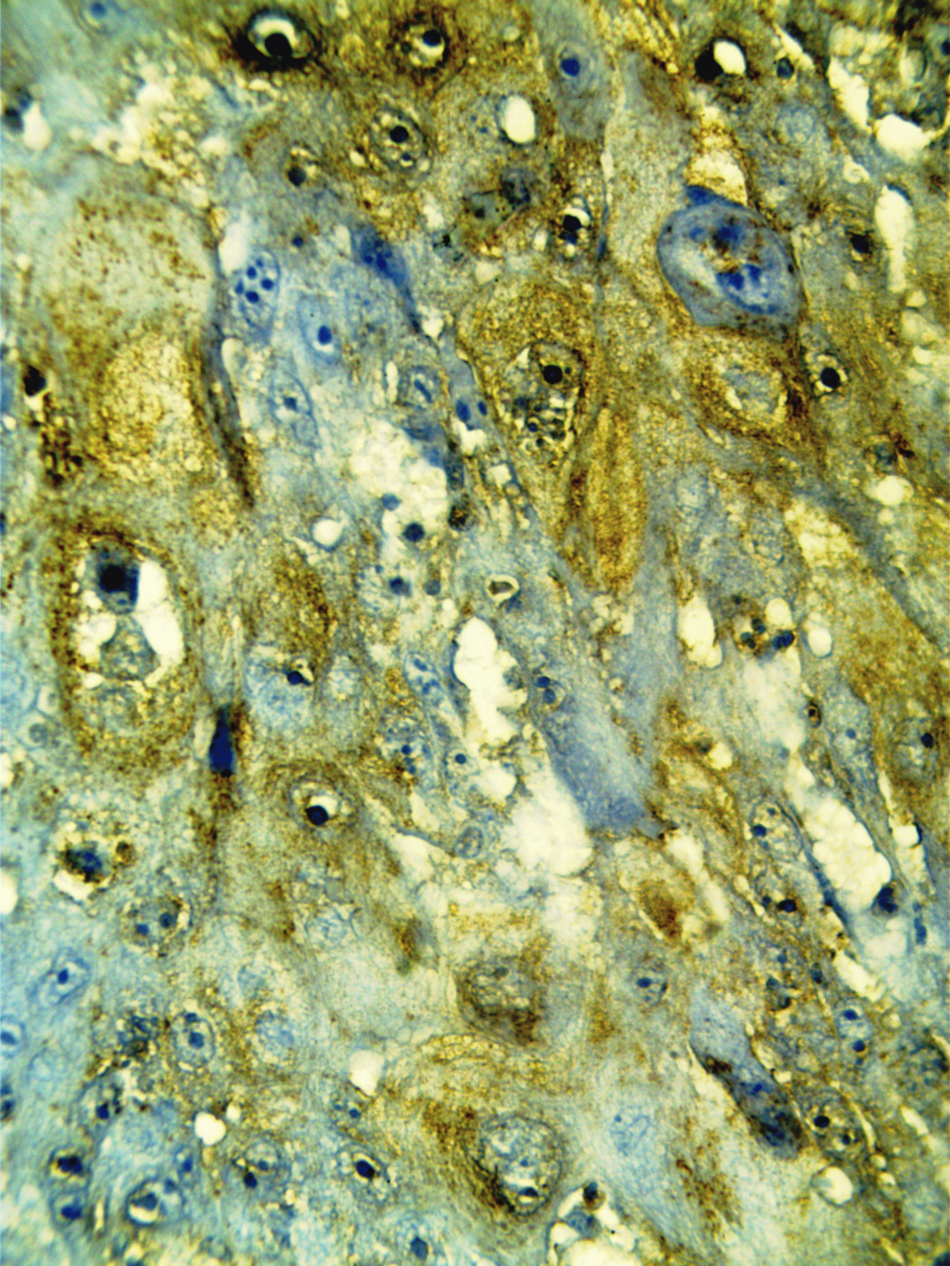
Photomicrograph showing positivity of CD68 (immunohistochemistry (IHC), 40x)

Immunohistochemistry was performed to confirm the origin and nature of the foamy cells which were strongly positive for CD68 (Figure 3) and Cathepsin-B establishing a macrophagic lineage.

**Figure 4:**
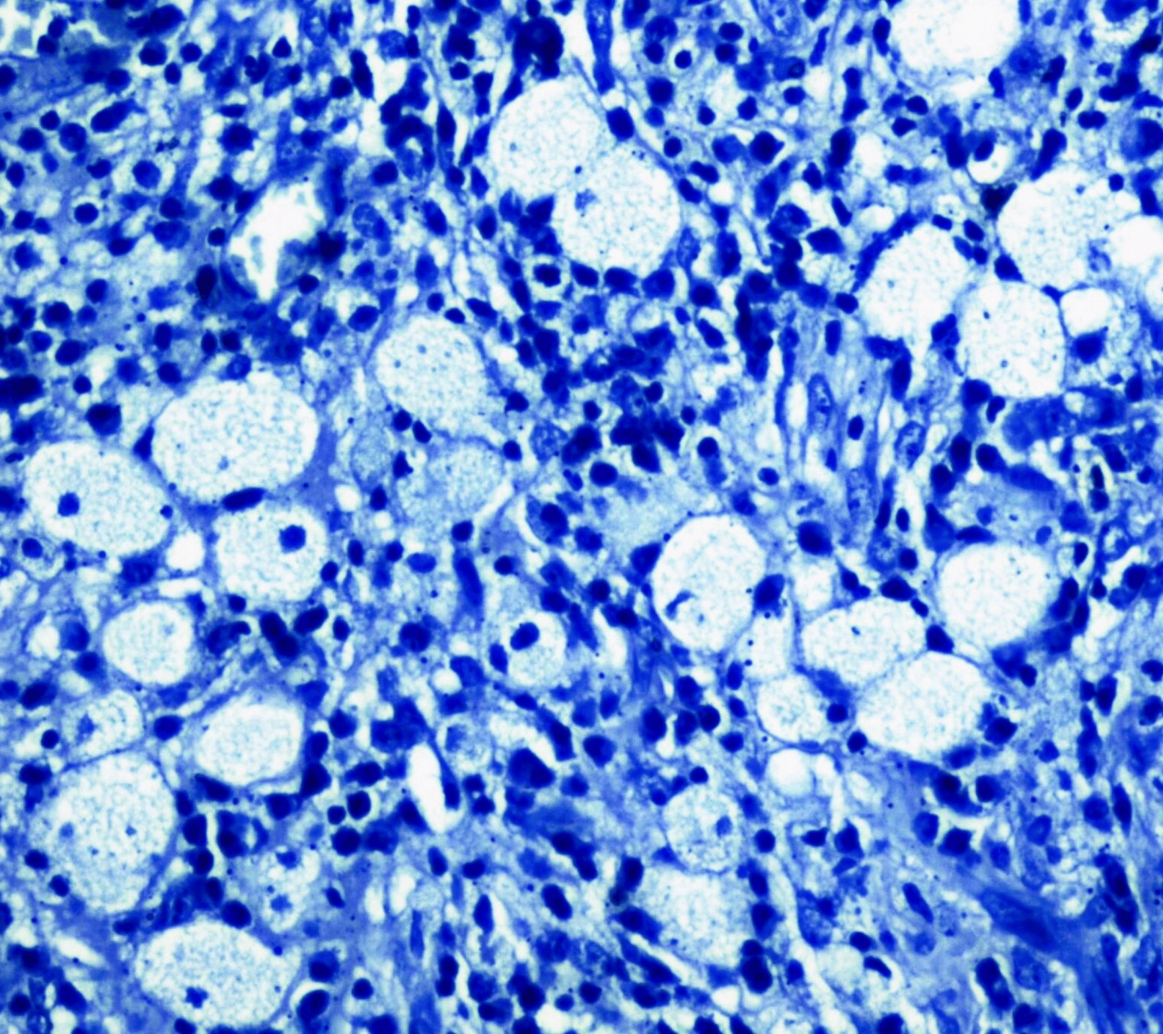
Photomicrograph of negative result to S100 (immunohistochemistry (IHC), 40x)

The foam cells were negative with S-100 (Figure 4).

**Figure 5:**
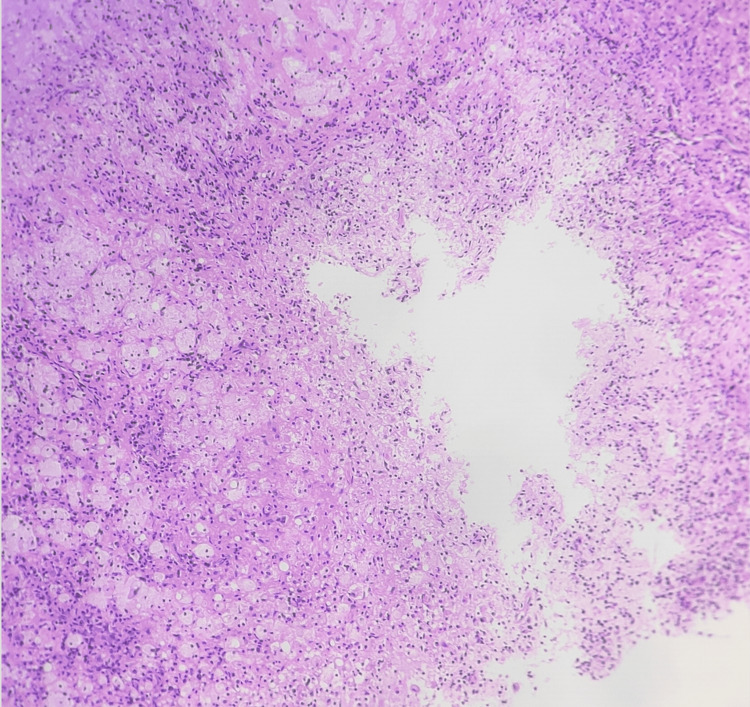
Photomicrograph showing xanthoma cells in the stroma, ulcerated epithelium along with inflammation (H&E, 4x) H&E: Hematoxylin and Eosin stain.

**Figure 6:**
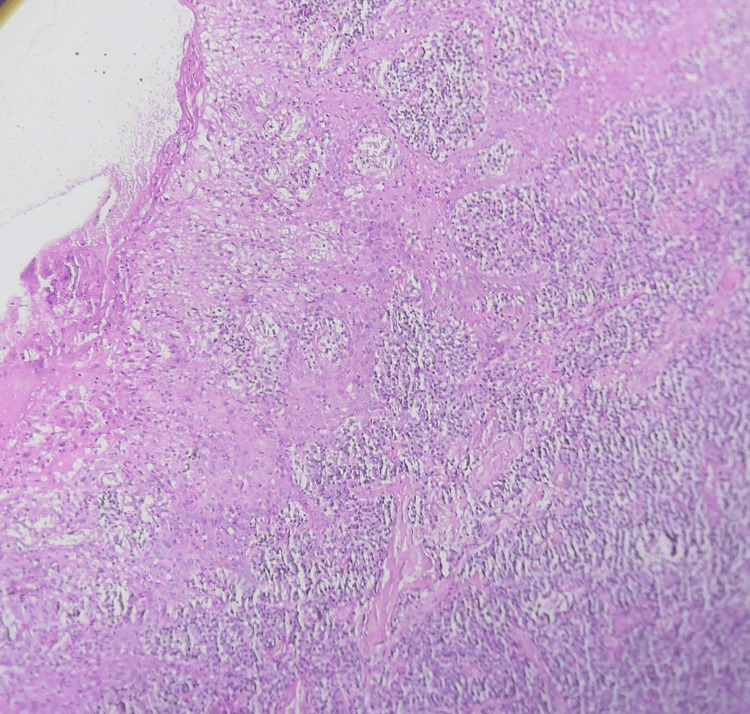
Photomicrograph showing hyperplastic parakeratinized stratified squamous epithelium and intense inflammation (H&E, 20x) H&E: Hematoxylin and Eosin stain.

**Figure 7:**
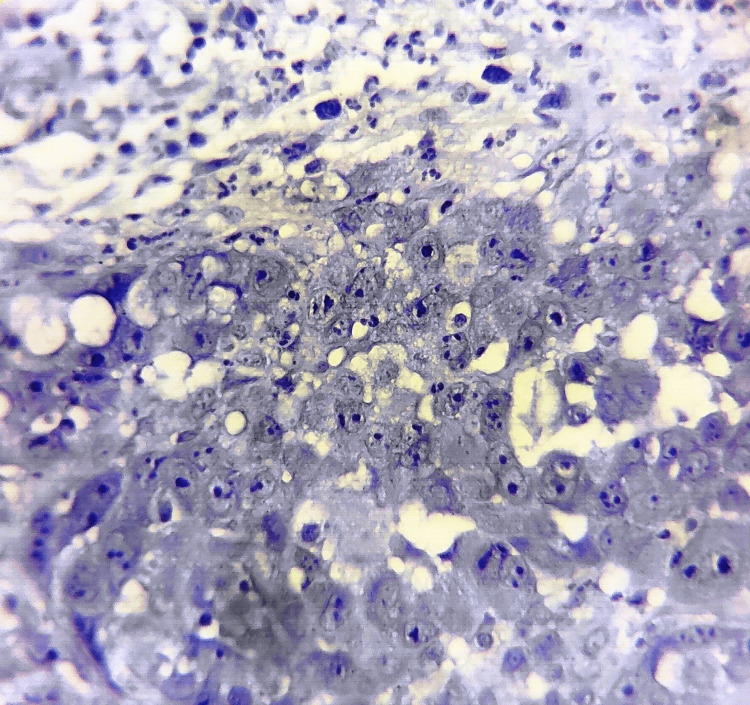
Photomicrograph of the xanthoma cells that were negative to PAS staining (PAS, 40x) PAS: Periodic acid-Schiff stain.

**Figure 8:**
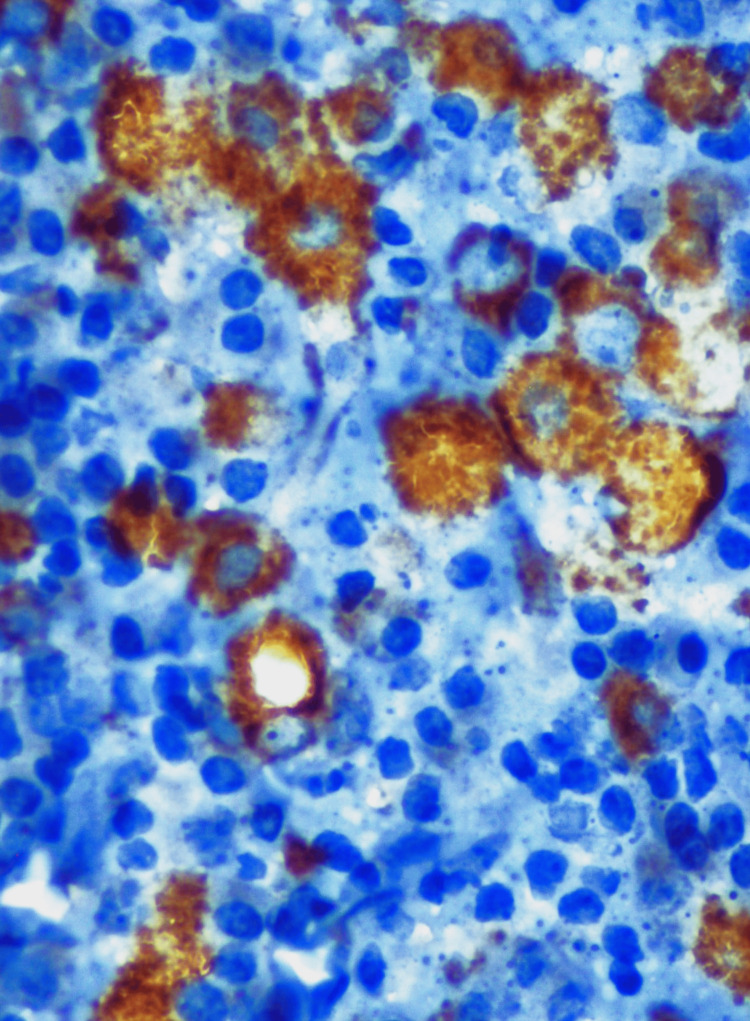
Photomicrograph showing Cathepsin-B positivity as brown stain under immunohistochemistry (IHC, 40x)

Considering overall features and results (Appendix, Figures 5-8), the final diagnosis was verruciform xanthoma of the lower lip. Eighteen months have passed since the excision and the patient is remaining disease-free on follow-up without any local recurrence.

## Discussion

Oral verruciform xanthoma (OVX) is a hyperplastic condition of the oral epithelium characterized by the presence of lipid-laden macrophages (foamy histiocytes) in the superficial stromal region, just beneath the epithelium [5]. This rare lesion predominantly affects males, with a mean age of onset around 50 years [6]. Although the etiology and pathogenesis of OVX remain unclear, several hypotheses have been proposed to explain its development.

One hypothesis suggests that the accumulation of lipids within macrophages results from the degeneration of epithelial cells. As these cells degenerate, they release lipid material that is then scavenged by macrophages, leading to the formation of the characteristic foamy cells seen in OVX. Another theory proposes that the presence of foamy cells affects the metabolism of the epithelial cells causing hyperkeratosis and leading to the verrucous and papillary architecture typical of OVX. Both hypotheses emphasize the importance of interactions between macrophages, keratinocytes, and chronic inflammation, which is supported by the observation of significant sub-epithelial chronic inflammation in many cases of OVX [3,7,8].

A genetic aspect of xanthoma formation has also been explored. A missense mutation in exon 6 of the 3β-hydroxysteroid dehydrogenase (NSDHL) gene, which is vital for cholesterol biosynthesis, has been identified in 22% of cutaneous xanthoma [9]. However, this mutation has not yet been studied in OVX. It is known that OVX, like cutaneous xanthomas, is not associated with human papillomavirus (HPV) infection [10]. The frequent occurrence of OVX on the masticatory mucosa has led to the hypothesis that local irritants, such as trauma or inflammation, may induce epithelial degeneration, with the resulting degenerated epithelium forming lipids that are then scavenged by macrophages [3]. This process is thought to contribute to the formation of foamy histiocytes and, subsequently, the development of OVX. However, this theory does not fully explain the presence of OVX in areas where trauma is not common, such as the soft palate or the floor of the mouth [11]. Additionally, microscopic examination often reveals no evidence of degenerative epithelial cells, and the persistence of foamy cells in the connective tissue further complicates this hypothesis.

Some researchers suggest that the epithelial changes observed in OVX may be secondary to the presence of foamy cells, or they might be "illusionary," resulting from the upward pressure exerted by the underlying macrophages. The wide distribution of OVX across various oral sites suggests that while trauma and irritation may play a role in its development, other factors, such as chronic inflammation or local immune responses, may also contribute to the occurrence of these lesions in less trauma-prone areas [5,6,11]. In clinical practice, OVX typically presents as a solitary lesion rather than multiple lesions, regardless of the site of occurrence. Clinical differential diagnoses include squamous papilloma, verruca vulgaris, fibroma, leukoplakia, and squamous cell carcinoma. Notably, men are more frequently affected by OVX than women, and the nodular form of OVX is more prevalent than the plaque or ulcerative forms. The surface texture of OVX is commonly granular and rough, contributing to its distinctive appearance [12].

Histopathologically, OVX exhibits unique features that are essential for its diagnosis. The lesion typically appears as a well-circumscribed area in the oral mucosa, with a verrucous or papillary surface architecture. The hallmark of OVX is the presence of numerous foamy histiocytes within the connective tissue, just beneath the epithelial layer. These foamy histiocytes are large, lipid-laden macrophages with foamy, vacuolated cytoplasm from the accumulation of lipid material. The nuclei of these cells are typically small, eccentric, and uniform. The presence of intracellular lipids can be confirmed by staining the cytoplasm with lipid-specific stains, such as Oil Red O or Sudan Black [13,14]. These foamy cells are usually localized to the superficial lamina propria, often forming clusters that closely approximate the overlying epithelium [14].

The overlying epithelium in VX may show a range of changes, most commonly parakeratosis and acanthosis, leading to the thickened, verrucous appearance of the lesion. The rete ridges of the epithelium often exhibit elongation and may appear to be pushed upward by the underlying xanthoma cells, contributing to the papillary architecture. Despite these changes, there is typically no evidence of cellular atypia or dysplasia in the epithelial layer. A chronic inflammatory infiltrate, predominantly composed of lymphocytes and plasma cells, is often present in the connective tissue surrounding the xanthoma cells. This inflammation further supports the hypothesis that chronic irritation or inflammation may play a role in the pathogenesis of VX [3,11,14].

VX has been reported in association with various other oral conditions, including oral lichen planus, graft-versus-host disease following bone marrow transplantation, oral squamous cell carcinoma, leukoplakia, amyloidosis in patients undergoing chemotherapy or radiotherapy for lymphoma, oral pemphigus vulgaris, oral discoid lupus erythematosus, and submucous fibrosis. Extraoral sites comprised the scrotum, vulva, cheek, wrist, gluteal region, and abdominal wall [6,11,15-18]. These associations suggest that VX may not only be a response to local irritation or trauma but may also be related to broader systemic conditions or treatments that affect the oral mucosa. Table 1 summarizes the reported literature.

**Table 1:** Table summarizing reported literature on verrucous xanthoma CHILD: congenital hemidysplasia with ichthyosiform nevus and limb defects.

Author	Year	Reported cases	Clinical findings
Belknap et al. [1]	2020	212	Gingiva, palate, tongue
Atarbashi-Moghadam et al. [2]	2021	1	Tongue
Oliveira et al. [4]	2001	4	Masticatory mucosa
Sah et al. [5]	2008	2	Buccal mucosa, lower labial mucosa
Philipsen et al. [6]	2003	282	Gingiva, masticatory mucosa
Dorankula et al. [8]	2013	1	Buccal mucosa
Getz et al. [9]	2019	16	8 samples showed NSDHL mutation associated with CHILD syndrome
de Andrade et al [10]	2015	20	Palate, buccal mucosa, gingiva
Tamiolakis et al. [11]	2018	429	Gingiva, palate, tongue, lower lip, alveolar mucosa
Capocasale et al. [12]	2017	1	Buccal mucosa and palate
Garcia et al. [13]	2016	1	Palate
Gannepalli et al. [14]	2019	1	Gingiva with oral submucous fibrosis
Javadi et al. [15]	2023	110	Palate, buccal mucosa, gingiva, tongue, and extra-oral sites
Kimura et al. [16]	2016	1	Lower gingiva
Preto et al. [17]	2024	2	Tongue, floor of the mouth
Shigeoka et al. [19]	2020	5	Gingiva, palate
Jia et al. [20]	2023	1	Tongue with oral lichen planus
Current case	2024	1	Lower lip: non-keratinized mucosa

Histopathology recognizes three types of OVX: Type A (verrucous type) with hyperparakeratosis, acanthosis, and elongated rete ridges, Type B (papillary type) with fingerlike projections of stratified squamous epithelium containing connective tissue cores, and Type C (flat type) with mild acanthosis, variable elongation of rete ridges and thin parakeratosis. The flat type is the most common [16]. As we found hyperplastic epithelium along with ulceration, we could group it as a Type C-flat type of OVX.

Preto et al. [17] have recommended that OVX in high-risk sites like the tongue and floor of the mouth should be monitored strictly. Kannan et al. [18] have discussed the diagnostic workflow for lower lip lesions and differential diagnosis. Shigeoka et al. [19] reported that CD168-positive macrophages were involved in the pathogenesis of OVX by regulating vascular endothelial growth factor (VEGF) expression. Jia et al. [20] reported a case of OVX with oral lichen planus. Recommended treatment is surgical excision, and the recurrence is rare [16]. Our patient also underwent complete excision and remained without any recurrence after 18 months.

## Conclusions

Oral verruciform xanthoma (OVX) is a rare, enigmatic condition with a distinct histopathological profile. While its exact etiology and pathogenesis remain unclear, the interaction between foamy histiocytes, epithelial cells, and chronic inflammation plays a central role in its development. OVX typically occurs in areas of the oral cavity exposed to trauma but can also be present in less trauma-prone regions, suggesting a multifactorial origin. Understanding the histopathology of OVX is crucial for accurate diagnosis and differentiation from other similar-appearing oral lesions.
